# Characterization of Transcription Regulatory Domains of OsMADS29: Identification of Proximal Auxin-Responsive Domains and a Strong Distal Negative Element

**DOI:** 10.3389/fpls.2022.850956

**Published:** 2022-04-25

**Authors:** Ridhi Khurana, Sanchi Bhimrajka, Gundra Sivakrishna Rao, Vibha Verma, Neelima Boora, Gautam Gawande, Meenu Kapoor, Khareedu Venkateswara Rao, Sanjay Kapoor

**Affiliations:** ^1^Interdisciplinary Centre for Plant Genomics and Department of Plant Molecular Biology, University of Delhi South Campus, New Delhi, India; ^2^Centre for Plant Molecular Biology, Osmania University, Hyderabad, India; ^3^University School of Biotechnology, Guru Gobind Singh Indraprastha University, New Delhi, India

**Keywords:** auxin, *cis*-elements, MADS-box, *Oryza sativa*, OsMADS29, seed development

## Abstract

OsMADS29 (M29) is a seed-specific MADS-box transcription factor involved in programmed cell death of nucellar tissue and maintaining auxin:cytokinin homeostasis. It affects embryo and endosperm development and starch filling during seed development in rice. Its expression seems to be tightly regulated by developmental, spatial, and temporal cues; however, *cis-* and *trans*-regulatory factors that affect its expression are largely unknown. *In silico* analysis of the 1.7 kb upstream regulatory region (URR) consisting of 1,290 bp promoter and 425 bp 5′-UTR regions revealed several auxin-responsive and seed-specific *cis*-regulatory elements distributed across the URR. In this study, the analysis of four URR deletions fused to a downstream β-glucuronidase (GUS) reporter in transgenic rice has revealed the presence of several proximal positive elements and a strong distal negative element (NE). The promoter regions containing auxin-responsive elements responded positively to the exogenous application of auxins to transgenic seedlings. The proximal positive elements are capable of driving reporter expression in both vegetative and reproductive tissues. In contrast, the NE strongly suppresses reporter gene expression in both vegetative and reproductive tissues. In a transient onion peel assay system, the NE could reduce the efficacy of a 2x CaMV 35S promoter by ∼90%. Our results indicate the existence of a complex array of positive and negative regulatory regions along with auxin-responsive elements guiding the development-dependent and spatial expression of *M29*.

## Introduction

The survival of living organisms is correlated with their ability to successfully reproduce and to sense, perceive, and adapt to diverse abiotic and biotic environmental cues. Each of these aspects requires a great deal of coordination between sensory mechanisms and mechanisms that control gene expression. Regulating developmental transitions requires transcribing the relevant genetic information at the right moment and spurring the correct phenotypic response (She et al., 2013; Ichihashi et al., 2020; Borg et al., 2021). Chromatin is the warehouse of genetic information, and its packaging is critical for both maintaining and transcribing this data.

Enhancer and silencer sequences located either upstream or downstream to the transcription start site (TSS) recruit transcription factors and their coactivators to mediate precise spatiotemporal expression of genes located either nearby or several kilobases away (Shlyueva et al., 2014; Weber et al., 2016). Concomitantly, a number of functional regulatory *cis*-elements have been identified in either intronic (Broeckling et al., 2016; Xie et al., 2018), exonic (Liu et al., 2017), or the untranslated regions (UTRs) (Wang and Chekanova, 2019). A single element present in a promoter may confer both positive and negative influence on the transcription, as observed in the case of *AB80* and *rbcS-3A* genes in Pea (Coruzzi et al., 1984; Simpson et al., 1986; Kuhlemeier et al., 1987). However, in other cases, combinatorial distribution of *cis*-acting elements exerting either a positive or negative control over the transcription of genes is crucial for fine-tuning gene expression networks. Transcriptome reprogramming is an essential factor in orchestrating developmental transitions (Deveshwar et al., 2011; Sharma et al., 2012; Yi et al., 2019).

In addition, phytohormones have also been found to be integral factors influencing transcriptome profiles for adapting to developmental or stress conditions (Nemhauser et al., 2006; Borah et al., 2017; Ma et al., 2019). Auxin accumulation patterns regulate various aspects of embryonic and postembryonic development. Studies on developing embryos in *Arabidopsis* (Friml et al., 2003) and maize (Chen et al., 2014) show characteristic auxin accumulation profiles. In rice, *de novo* indole-3-acetic acid (IAA) production in the young embryos is predicted to rely on the TAR/YUCCA pathway supported by increased expression of *OsTAR2, OsYUC7, OsPIN1a, OsIAA1, 10, 14, 15, 19, 20*, and *24* genes (Uchiumi and Okamoto, 2010; Abu-Zaitoon et al., 2012; Itoh et al., 2016), indicating a significant role of auxin in rice embryo and endosperm development. Auxin is known to regulate the transcription of target genes both positively and negatively *via* the auxin response factors (ARFs), Aux/IAA proteins, and the 26S proteasome machinery (Lavy and Estelle, 2016; Powers and Strader, 2020).

After fertilization, the development of the zygote into a multicellular seed requires a coordinated interplay of multiple gene regulatory networks (Abiko et al., 2013; Anderson et al., 2017; Deushi et al., 2021). To understand the complexity of transcriptomic alterations that occur after fertilization in developing embryos, we need to understand the mechanisms that regulate gene expression. OsMADS29 (M29) is a seed-specific MADS-box transcription factor that has been implicated in embryo and endosperm development in rice. The expression of *M29* is known to be tightly regulated at the transcriptional, posttranscriptional, and posttranslational levels (Yin and Xue, 2012; Nayar et al., 2013, 2014). In preliminary studies involving the culture of detached ovaries from pollinated and unpollinated flowers and their subsequent treatment with IAA and 2,4-dichlorophenoxyacetic acid (2,4-D), the expression of *M29* was shown to be induced after pollination as well as upon treatment with exogenous auxin, suggesting the involvement of auxins in the transcriptional regulation of M29 (Yin and Xue, 2012). Therefore, understanding the transcriptional regulatory domains of M29 would help shed light on the regulation of rice seed development.

In this study, we have analyzed the upstream regulatory region (URR) of M29 to identify domains that impart precision to its spatiotemporal and developmental expression. The serial URR deletions were created based on the presence of several seed-specific and auxin-responsive elements and analyzed for their ability to drive reporter gene expression in transgenic rice. The experiments reveal the presence of a proximal positive, auxin-responsive regulatory region along with a strong negative element in the distal region of the URR. Our data provide insights into a complex interplay of negative and positive *cis*-elements used by biological systems for the precise regulation of gene expression at the transcription level.

## Materials and Methods

### Prediction of Conserved *cis*-Elements in M29 Upstream Regulatory Region

The 1.5 kb upstream and 0.4 kb downstream regions of OsMADS29 were downloaded from RAP-DB (Rice Annotation Project, 2007) and analyzed using the PLACE database (Higo, 1998) motif list in CLC Main Workbench version 7.0. A frequency distribution curve was made using Microsoft Excel.

### Cloning Upstream Regulatory Region Deletions

The –1290..425 region of M29 was PCR amplified from genomic DNA from Indica rice using gene-specific primers (Sigma Aldrich-Merck, United States) and Phusion™ High-Fidelity DNA Polymerase (Thermo Fisher Scientific, United States). The remaining three deletions were PCR amplified from this fragment (–618..425, –355..425, and –78..425). These fragments were mobilized into the pENTR™/D-TOPO™ entry vector and validated by restriction fragment analysis and DNA sequencing. From the entry clones, these constructs were mobilized into destination plant transformation vector pMDC164 harboring a glucuronidase (GUS) reporter *via* LR Clonase II Enzyme Mix (Thermo Fisher Scientific, United States). The destination vector clones were validated by restriction fragment analysis.

### Cloning Negative Element

For the control vector, 2x CaMV 35S was PCR amplified from the pB4NU vector using promoter-specific primers and Phusion™ High-Fidelity DNA Polymerase and mobilized into the pENTR™/D-TOPO™ entry vector. The 2x CaMV 35S was further mobilized into two destination vectors, pMDC110 and pGWB653, which harbor green fluorescent protein (GFP) and red fluorescent protein (RFP) reporters, respectively, *via* LR Clonase II Enzyme Mix (Thermo Fisher Scientific, United States). After validation by restriction fragment analysis, the 2x CaMV 35S:GFP:NosT cassette from the pMDC110 vector was cloned into the pBSK+ vector *via Eco*RI and *Not*I FastDigest™ restriction enzymes (Thermo Fisher Scientific, United States). Sequentially, the 2x CaMV 35S:RFP:NosT cassette was PCR amplified using primers containing the *Eco*RI restriction enzyme site and cloned into the final pBSK+ vector. The negative element region, as well as the four deletions (−1,290..–619, –1,103..–619, –956..–619, –845..–619, and –730..–619), was PCR amplified from the –1290..425 fragments. These primers contained the *Not*I restriction enzyme site at the 5′ end to facilitate cloning directly upstream of the 2x CaMV 35S:GFP:NosT cassette *via* the *Not*I restriction enzyme. All positive clones were validated *via* restriction fragment analysis and DNA sequencing.

### Transformation of *Agrobacterium tumefaciens* EHA105

*Agrobacterium tumefaciens* EHA105 was transformed with pMDC164 clones using electroporation. Electroporation was performed using the MicroPulser™ electroporation apparatus (Bio-Rad Laboratories, United States) as per the protocol mentioned in the user manual.

### Agrobacterium-Mediated Transformation of Indica Rice

*Agrobacterium tumefaciens* EHA105 clones harboring promoter:GUS fusion constructs in pMDC164 (Curtis and Grossniklaus, 2003) were used to transform the Indica rice variety IET 10364 (Toki et al., 2006).

### Copy Number Estimation of Transgenics by Southern Hybridization

Genomic DNA was isolated from mature leaf tissues of transgenic and untransformed wild-type (WT) Indica rice plants using the Cetyltrimethyl ammonium bromide (CTAB) method (Tapia-Tussell et al., 2005). At 37°C for 16 h, 15 μg of genomic DNA was digested by 10 μl of *Hin*dIII FastDigest™ enzyme (Thermo Fisher Scientific, United States). The digested fragments were resolved on a 0.8% agarose gel and transferred onto N + nylon membranes (Amersham Biosciences) *via* the capillary transfer method. The hybridized membrane was fixed by exposing it to UV (1200 J for 60 s) in a UV cross-linker. The hygromycin probe was radiolabeled with α-32P dCTP using the Random Primer Labeling Kit (BRIT). The membrane was processed further as per the manufacturer’s instructions.

### Analysis of Glucuronidase Reporter Expression

Tissue samples were first treated with 90% ice-cold acetone on ice for 3 min. Afterward, the samples were washed twice with GUS buffer without X-Gluc (0.1% Triton X-100, 10% methanol, 0.5 mM potassium ferrocyanide, 0.5 mM potassium ferricyanide, 10 mM EDTA pH 8.0, and 50 mM sodium phosphate buffer pH 7.0) (Jefferson, 1987). Finally, samples were treated with GUS buffer supplemented with 1 mM X-Gluc, and the buffer was vacuum-infiltrated for 15 min at room temperature. The samples were incubated at 37°C for 16 h.

### Fluorometric Quantitation of Glucuronidase Reporter Expression

Crude protein extracts were isolated from 50 to 100 mg transgenic and WT samples using 1 ml GUS extraction buffer (50 mM sodium phosphate buffer pH 7.0, 10 mM β-mercaptoethanol, 10 mM sodium EDTA pH 8.0, 0.1% sodium lauryl sarcosine, and 0.1% Triton X-100) (Jefferson, 1987). The total protein concentration of these crude extracts was measured using Bradford’s assay. A uniform protein amount of 6 μg was used from all samples for this assay. A total of 500 μl GUS extraction buffer (GUS assay buffer supplemented with 1 mM 4-methylumbelliferyl β-D-glucuronide; MUG) was added and mixed with 6 μg protein in 50 μl GUS extraction buffer. The samples were incubated at 37°C overnight (precisely 16 h). The reactions were stopped by adding 900 μl of 0.2 M sodium carbonate solution to 100 μl of the MUG reaction. The fluorescence intensities of all samples were measured using the TECAN M200 Infinity Pro microplate reader with the excitation set at 365 nm and the emission at 455 nm.

### Auxin Induction Assay

The 7-day-old seedlings were treated with 50 μM IAA supplemented in RGM and incubated for 1 and 3 h (Jain and Khurana, 2009). Mock treated samples were used as controls. GUS activity posttreatment was measured in terms of the accumulation of μM of 4-MU/mg protein/h.

### Transient Expression by Particle Bombardment

Particle bombardment was performed using the Biolistic PDS-1000/He particle delivery system (Bio-Rad, United States) according to an earlier described protocol (Lee et al., 2008) with a few minor modifications. For each construct, 2 μg of DNA was coated on 1 mg (0.5 mg used per shot) of 1 μm gold particle. The shooting parameters were 27 mm Hg vacuum, 1,100 psi helium pressure, with the target distance set at 6 cm. The plates were then sealed and incubated in the dark at room temperature for 12–14 h. The onion peels were then observed for GFP and RFP under a Leica SP5 confocal laser scanning microscope. For each construct, 12–15 images were captured with the gain set at constant for both the GFP and RFP channels.

### Fluorescence Intensity Measurement and Data Analysis

The images were exported to the Leica Application Suite X (LASX) for analysis of the fluorescence intensity. The images were opened in the Quantify section and a small ROI was drawn in the nucleus of the cell while avoiding the nucleolus. Several datasets were provided, out of which the mean value of the intensity was recorded for both the channels (GFP and RFP). The GFP/RFP ratio was calculated for each cell, and the average value was calculated using Microsoft Excel. The scatter plot to depict the GFP/RFP ratios was generated using the PlotsOfData online tool (Postma and Goedhart, 2019). The images were then edited and compiled in Adobe Photoshop CC 2018 and Adobe Illustrator CC 2018, respectively.

## Results

### OsMADS29 Upstream Region Has Conserved Seed-Specific and Auxin-Responsive Elements

**FIGURE 1:**
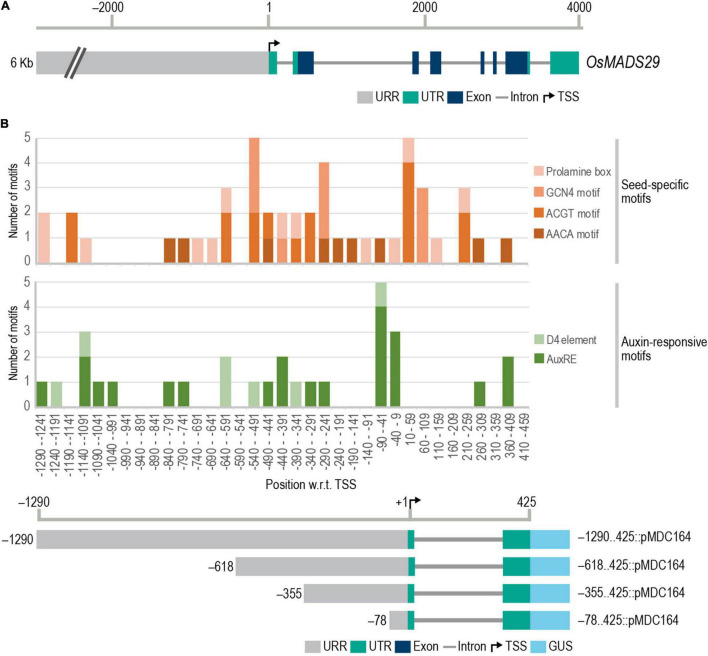
Gene structure of M29 and frequency distribution of conserved *cis*-elements in M29 URR. **(A)** Gene structure of M29 and its upstream region (chr02:3843135.3833130). **(B)** Frequency distribution of conserved seed-specific and auxin-responsive *cis*-elements in the M29 URR between –1,290 and 425 nt. In **(B)**, the *Y*-axis represents the number of motifs, and the *X*-axis represents 50 bp intervals of the M29 URR from –1,290 to −459 and at the bottom, a diagrammatic representation of the four deletions of the *M29* URR in the pMDC164 vector showing the 5′ and 3′ boundaries of each construct fused with the GUS reporter. Motifs were predicted using the PLACE database motif list in CLC Main Workbench version 20.0.4 and plotted using a stacked column graph in Microsoft Excel.

To identify *cis*-regulatory elements in the *M29* upstream region, the sequence of upstream and downstream regions to the TSS was downloaded from the Rice Annotation Project Database (RAP-DB) (Rice Annotation Project, 2007). For up to 6.3 kb upstream of M29, there is no other predicted gene (Figure 1A). Using the PLACE database (Higo, 1998) motif list and CLC Main Workbench version 20.0.4, the entire 6.3 kb upstream region, as well as the 5′UTR up to 425 bases downstream to the TSS, was scanned for the presence of conserved *cis*-elements. Considering the seed-specific expression of M29 and the role of auxin in plant embryogenesis, we narrowed our search to seed-specific and auxin-responsive elements. We screened for the presence of four seed-specific motifs, namely, the prolamin box, AACA motif, ACGT motif, and GCN4 motif (Foster et al., 1994; Onodera et al., 2001; Qu et al., 2008; Wu et al., 2015), and three auxin-responsive elements. Auxin-responsive motifs may either be a singular motif comprising a 6 bp core (TGTCTC) or a composite motif comprising a coupling element along with the 6 bp core. The D1 and D4 elements are composite auxin-responsive elements identified in the *GmGH3* promoter (Ulmasov et al., 1995). Various other composite element combinations have been predicted using bioinformatic tools but have not yet been experimentally validated (Berendzen et al., 2012; Mironova et al., 2014; Cherenkov et al., 2018). We have used only the experimentally validated auxin-responsive elements, namely, the 6 bp core, D1, and D4 (Ulmasov et al., 1995).

Our analysis revealed multiple seed-specific and auxin-responsive elements in the proximal and distal promoter regions (Figure 1B). We observed a high density of the four seed-specific elements, the auxin-responsive core, and the D4 composite element. No D1 elements were found in our analysis. Based on the distribution of these *cis*-elements, we selected four sites in the *M29* URR, *viz.*, –1290, –618, –355, and –78, for making serial deletions for reporter gene expression analyses in transgenic rice (Figure 1B). These sites were carefully chosen to avoid any truncation of auxREs.

### Characterization of Upstream Regulatory Region: Glucuronidase Fusions in Transgenic Rice

**FIGURE 2:**
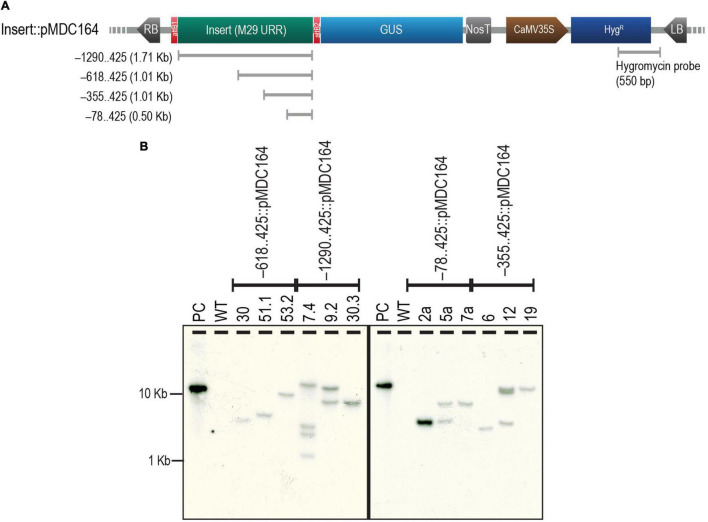
Analysis of T-DNA copy number of transgenics by Southern hybridization. **(A)** Map of pMDC164 showing the T-DNA region harboring the URR deletion inserts fused to a downstream GUS reporter and the CaMV 35S promoter-driven hygromycin antibiotic-selection gene. The region of hygromycin and the vector backbone used as a probe for the Southern hybridization experiments has been indicated. **(B)** Results of the Southern hybridization experiment show the copy number in each of the transgenics. Positive control (PC) is a *Hin*dIII linearized –78..425:pMDC164 plasmid and an untransformed wild-type (WT) was used as a negative control for this experiment. The blot was photographed using an HP Scanjet G4050 scanner.

The four selected regions of the *M29* URR, i.e., –1290..425, –618..425, –355..425, and –78..425, were cloned upstream to a GUS reporter in the pMDC164 backbone (Supplementary Figure 1). Three independent transgenic lines from each of the four constructs were analyzed for T-DNA copy number by southern hybridization experiments using a hygromycin-specific probe (Figure 2A). We found two single-insertion lines for –618..425, –355..425, and –78..425; however, we could detect only one single-insertion line for –1290..425 (Figure 2B). We chose to use two single-insertion lines for the –618..425, –355..425, and –78..425 constructs for the detailed reporter expression analysis in rice. However, for the –1290..425 construct, along with a line that was validated for single insertion, we used a double-insertion line as this line also exhibited identical GUS expression. In the case of the –78..425 construct, only one of the two single insertion lines bore seed; the other did not. Therefore, we depicted data from only one line for the –78..425 construct. In other constructs, both the lines analyzed exhibited similar expression patterns, and the data from one of those lines is shown here. No phenotypic variation was observed in the transgenic plants when compared to the untransformed control WT plants (Supplementary Figure 2), and there was no phenotypic difference within the lines tested for different constructs.

For the GUS reporter gene expression analysis in rice, we chose 15 tissues representing different developmental stages; root and shoot from 7-day-old seedlings, and the mature plant, leaf blade, leaf sheath, intercalary meristem, and ten seed stages, i.e., gynoecium of mature floret (MF) (GyMF) and 0, 1, 2, 4, 6, 8, 10, 14, and 20 days after pollination (DAP). WT untransformed plants were used as a negative control for these analyses. The entire GUS expression profiling has been performed on samples from the T2 generation of transgenic plants.

### Analysis of Glucuronidase Reporter Expression in Early Stages of Seed Development

**FIGURE 3:**
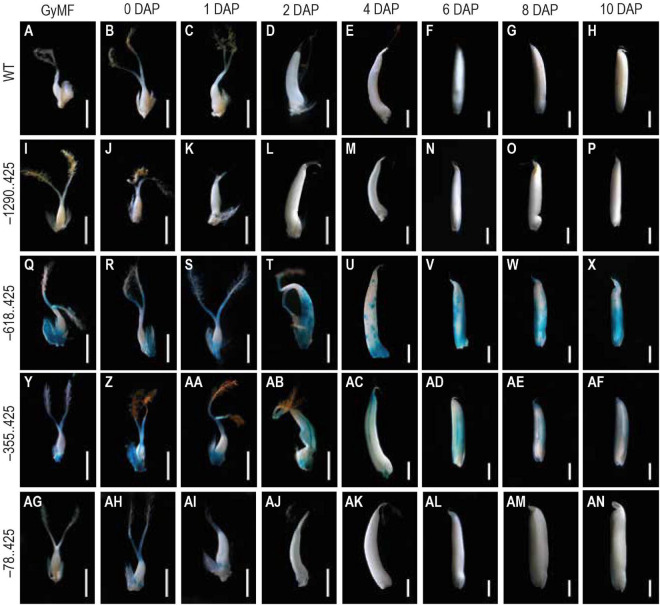
Analysis of GUS expression during early seed stages starting from unfertilized floret to 10 DAP stage in WT and transgenics. The panels show whole mounts of GUS-stained gynoecium of mature floret (GyMF) and seed stages from 0 to 10 DAP in **(A–H)** WT, **(I–P)** –1290. 425, **(Q–X)** –618. 425, **(Y–AF)** –355. 425, and **(AG–AN)** –78. 425. Samples were photographed using a stereozoom dissecting microscope. Scale bar in GyMF; 0, 1, 2, and 4 DAP samples represents 1 mm; 6, 8, and 10 DAP samples represents 2 mm.

For analyzing the GUS reporter expression in our transgenics, we examined 10 different seed stages, beginning from GyMF to 20 DAP. Here, GyMF represents the gynoecium before pollination, and the remaining stages represent the seed as DAP. GUS expression was not visible in any of the seed samples from our longest construct –1290..425 (Figures 3I–P), similar to that of the WT untransformed control seed samples (Figures 3A–H). GUS expression was observed in our second and third constructs, –618..425 (Figures 3Q–X) and –355..425 (Figures 3Y–AF) during the seed stages. Both of these seed samples show similar GUS expression in the GyMF and 0–2 DAP seeds, where GUS is observed in the style, lodicules, and at the base of the ovary and expands to the pericarp by 2 DAP. GUS accumulates over the pericarp in both –618..425 and –355..425 seeds from 4 to 10 DAP. Between 4–10 DAP, GUS was visible in the embryo of –618..425 seeds (Figures 3U–X) and not the –355..425 seeds (Figures 3AC–AF). In our fourth and smallest constructs, –78..425 GUS expression was observed in the style, lodicules, and in the anther filaments (Figures 3AG–AI) (filaments got cut while dissecting seeds; the remnants can be seen toward the base of the ovary). No GUS expression was observed in the –78..425 seeds from 2–10 DAP (Figures 3AJ–AN).

### Analysis of Glucuronidase Expression in Late Stages of Seed Development

**FIGURE 4:**
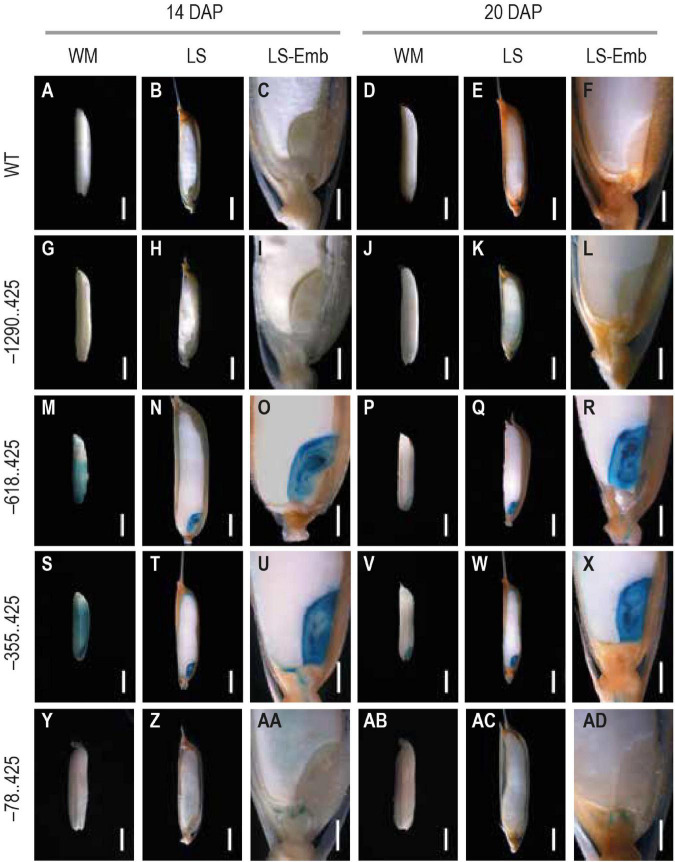
Analysis of GUS expression in mature seed stages 14 and 20 DAP from WT and transgenic plants. The panels show whole mounts (WM), hand-cut longitudinal section (LS), and a magnification of the LS showcasing the embryo (LS-Emb) of mature 14 and 20 DAP seeds from **(A–F)** WT, **(G–H)** −1290..425, **(M–R)** −618..425, **(S–X)** −355..425, and **(Y–AD)** −78..425 rice transgenics. Samples were photographed using a Leica Mz12.5 Stereozoom microscope. The scale bar in 14 and 20 DAP WM, and LS samples represents 2 mm and in LS-Emb samples is 0.5 mm.

We chose two mature seed stages, 14 and 20 DAP, for analyzing GUS reporter expression. GUS expression was not visible in either the untransformed control (Figures 4A–F) or the –1290..425 seed samples (Figures 4G–L) at either of these developmental timepoints. GUS expression was observed in the pericarp and the embryo in both the –618..425 and –355..425 seed samples (Figures 4M–O,S–U) at 14 DAP. At the final stage, GUS expression was restricted to the embryo at the 20 DAP and was no longer visible in the pericarp in both constructs (Figures 4P–R,V–X). In the embryo at 14 and 20 DAP, the GUS-derived blue color could be seen across the entire embryo, in the scutellum, the coleoptile, the plumule, and the radicle (Figures 4O,R,U,X, respectively). GUS expression was observed in the endosperm of the 14 DAP seed (Figures 4Y–AA) and not in any other portion of the developing seed in the –78..425 construct samples (Figures 4AB–AD).

**FIGURE 5:**
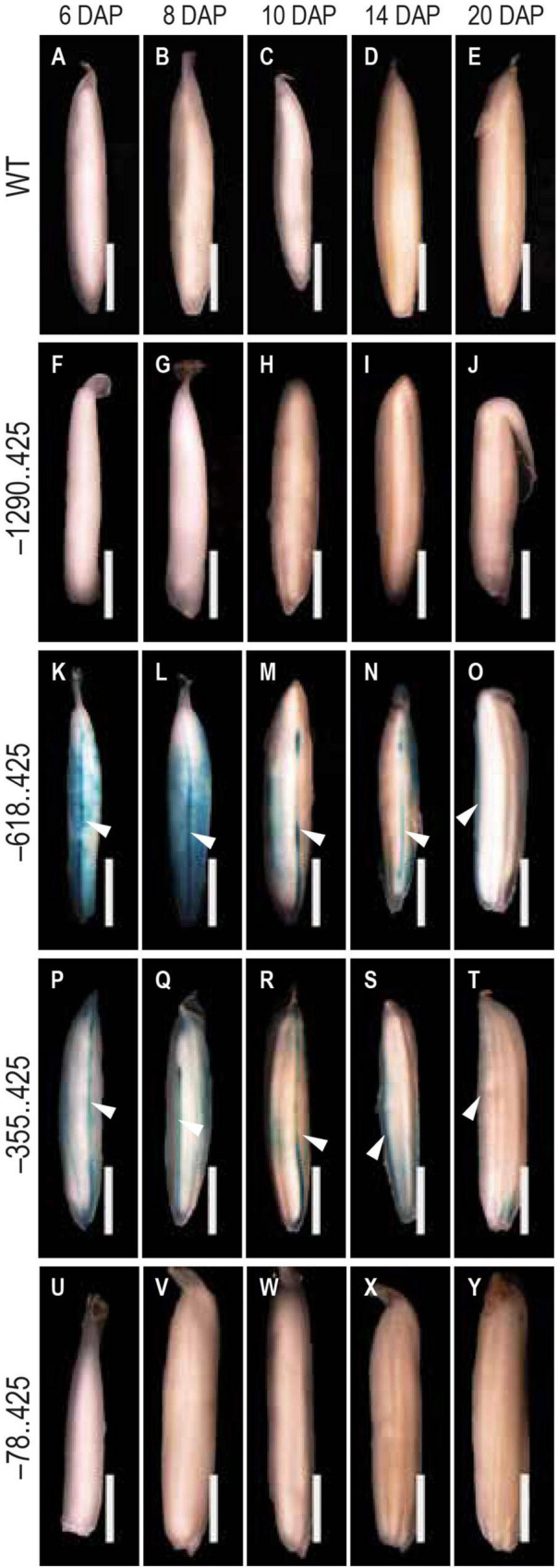
Analysis of GUS expression pattern in the dorsal vascular trace. The panels show the GUS expression patterns in the dorsal vascular trace of 6–20 DAP seeds **(A–E)** WT, **(F–J)** –1290..425, **(K–O)** –618..425, **(P–T)** –355..425, and **(U–Y)** –78..425. White arrowheads in **(K–T)** indicate the dorsal vascular trace. Samples were photographed using a Leica Mz12.5 Stereozoom microscope. The scale bar represents 2 mm.

**FIGURE 6:**
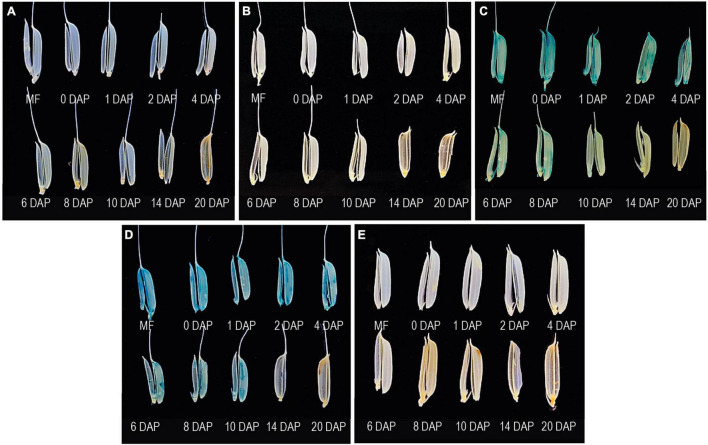
Analysis of GUS expression in the lemma and palea in WT and transgenic plants beginning from the mature floret (MF) and post-fertilization seed development stages in 0–20 DAP. The panel shows the GUS accumulation patterns in the lemma and palea of **(A)** WT, **(B)** –1290..425:pMDC164, **(C)** –618..425:pMDC164, **(D)** –355..425:pMDC164, and **(E)** –78..425:pMDC164 transgenics. Samples were photographed using phone camera.

During 6–20 DAP, GUS expression was visible in the dorsal vascular trace in –618..425 and –355..425 seeds (Figures 5K–O,P–T). Similar GUS expression was not observed in the untransformed control (Figures 5A–E), –1290..425 (Figures 5F–J), and –78..425 (Figures 5U–Y) seed tissues examined. GUS expression was also observed in the lemma and palea in the MF and seeds from 0 to 10 DAP in –618..425 (Figure 6C) and –355..425 (Figure 6D). GUS accumulation is prominent in the lemma and palea up to 8 DAP and subsides by 10 DAP, and no GUS expression was visible in the lemma and palea at 14 and 20 DAP (Figures 6C,D). GUS expression was not observed in the lemma and palea of any of the seed stages analyzed in either untransformed control (Figure 6A), –1290..425 (Figure 6B), and –78..425 (Figure 6E) samples.

### Analysis of Glucuronidase Reporter Expression in Vegetative Stages

**FIGURE 7:**
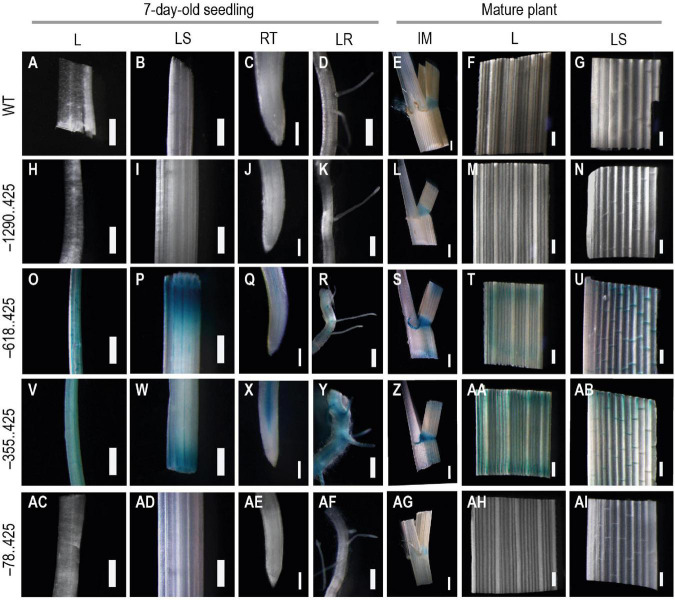
Analysis GUS expression in vegetative tissues from young and mature WT and transgenic plants. The panel shows whole mounts of GUS-stained leaf (L), leaf-sheath (LS), root tip (RT), and lateral root (LR) from 7-day-old seedling and intercalary meristem (IM), leaf (L), and leaf-sheath (LS) from mature plants, **(A–G)** WT, **(H–N)** −1290..425, **(O–U)** −618..425, **(V–AB)** −355..425, and **(AC–AI)** −78..425 transgenic rice plants. Samples were photographed using a Leica Mz12.5 Stereozoom microscope. In 7-day-old-seedling samples, the scale bar represents 0.5 mm in L, LS, and LR samples and 0.2 mm in RT, in samples from mature plant 2 mm in IM, and 0.5 mm in L and LS.

GUS expression was not visible in any of the samples from the untransformed control plants, except for the intercalary meristem tissue (Figures 7A–G). The –1290..425 samples mirrored the untransformed plants with faint positive GUS signals observed only in the intercalary meristem region and in no other tissue (Figures 7H–N). There have been reports of GUS activity from various young tissues of untransformed *Arabidopsis*, *Brassica*, tobacco, and rice plants (Sudan et al., 2006; Arul et al., 2008). Similar GUS expression was visible in all vegetative tissues examined for –618..425 (Figures 7O–U) and –355..425 (Figures 7V–AB). In samples from both of these constructs, GUS expression was observed in leaves, leaf sheaths, and in the vascular bundles of the root tip and the lateral root of young 7-day-old transgenic seedlings (Figures 7O–R,V–Y, respectively). Reporter expression was also observed in the veins of mature leaves and leaf sheaths (Figures 7S–U,Z–AB, respectively). In the intercalary meristem samples, GUS was observed in the intercalary meristematic region (at the leaf and leaf-sheath junction) and the auricle (Figures 7S,Z). This expression pattern was very different from that observed in either the untransformed control plants or the –1290..425 transgenics (Figures 7E,L, respectively). In the samples from –78..425, low-level GUS expression was observed in the intercalary meristem zone between the leaf and the leaf sheath (Figure 7AG) similar to that observed in untransformed control plant samples with no GUS expression in the auricle (Figure 7E). No GUS expression was observed in the remaining vegetative tissues examined for the –78..425 construct (Figures 7AC–AF,AH–AI).

**FIGURE 8:**
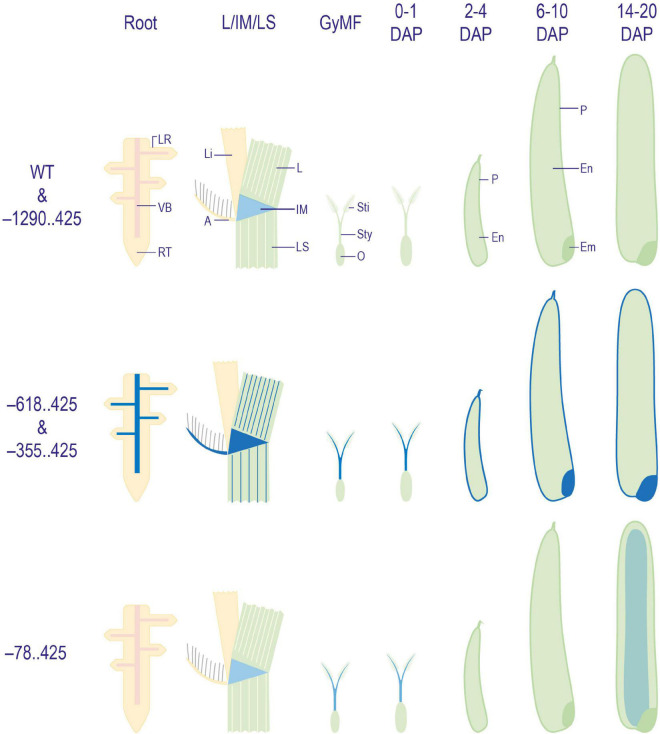
Diagrammatic representation of GUS expression patterns obtained in WT and transgenic plant tissues. Illustrations depicting a summary of patterns of GUS expression obtained in WT, and –1290..425, –618..425, –355..425, and –78..425 transgenic plants. Here, GyMF, gynoecium of mature floret; DAP, days after pollination; LR, lateral root; VB, vascular bundle; RT, root tip; L, leaf; Li, ligule; A, auricle; IM, intercalary meristem; LS, leaf sheath; Sti, stigma; Sty, style; O, ovary; P, pericarp; En,- endosperm; Em, embryo. The diagrams have not been drawn to scale.

The reporter gene expression profiling in all four URR:GUS fusion constructs in rice revealed three patterns. The first pattern, seen in –1290..425:pMDC164 lines, was similar to untransformed control WT plants, where the reporter gene expression was visible in neither the vegetative nor reproductive tissues studied. However, there was a slight non-specific GUS in the intercalary meristem region. The second pattern was observed in the –618..425:pMDC164 and –355..425:pMDC164 lines. In these lines, the expression was similar to that of the native *M29* (Yin and Xue, 2012; Nayar et al., 2013) in seed stages, but unlike in WT, the GUS expression in these URR deletions was also observed in vegetative stages. The –78..425:pMDC164 lines exhibited the third pattern where the reporter gene expression was observed only in the mature endosperm, specifically at the 14 DAP stage, unlike in any other construct analyzed. These GUS reporter patterns are summarized in Figure 8. To ensure that the lack of GUS expression in the –1290..425 construct in either vegetative or reproductive tissues was not a result of any cloning error, we got the entire 1.7 kb insert sequenced but found no conflicts with the reference sequence on RAP-DB (Rice Annotation Project, 2007), indicating that the lack of GUS activity might arise from the inability of the –1290..425 region to drive the expression of a downstream reporter gene.

### Fluorometric Estimation of Reporter Activity in Transgenic Plants

**FIGURE 9:**
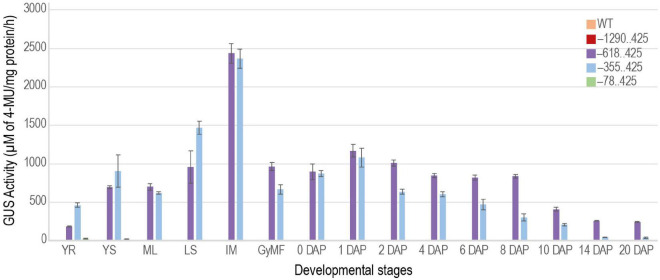
Quantitative measurement of GUS activity during different stages of development. Bar graph showing GUS activities obtained for –1290..425, –618..425, –355..425, and –78..425 constructs. The GUS activity measured in the WT was used as a control. YR and YS, young root and young shoot from 7-day-old light-grown seedlings; ML, mature leaf; LS, leaf sheath; IM, intercalary meristem from ∼ 60-days-old plants. Reproductive stages are represented by GyMF, gynoecium of mature floret; and seeds from 0–20 days after pollination (DAP) stages. The GUS activity is represented as μM of 4-MU/mg protein/h. Data represent information obtained from three biological replicates. Error bars represent the standard error.

To better understand and quantify the transcriptional strength of the URR deletions, we measured GUS activity in transgenic plants using the MUG assay (Jefferson, 1987; Figure 9). GUS activity was not observed for the –1290..425 construct besides the slight activity in the intercalary meristem similar to WT plants. The expression profiles of –618..425 and –355..425 URRs in seed tissues mimicked that of the native *M29* (Yin and Xue, 2012; Nayar et al., 2013), suggesting that these two regions contained necessary *cis*-elements to drive post-fertilization expression of downstream sequences in seeds. However, the added expression in vegetative tissues suggested the presence of suppressor/negative elements in the native URR that prevented its expression in vegetative tissues. The lowest GUS activity was observed in the –78..425 construct where weak signals were obtained in the 14 DAP seed samples and none in any other tissue sample tested. Three biological replicates were used for each sample for the fluorometric quantitation of GUS activity.

### Induction of M29 Upstream Regulatory Region Activity by Exogenous Application of Indole-3-Acetic Acid

**FIGURE 10:**
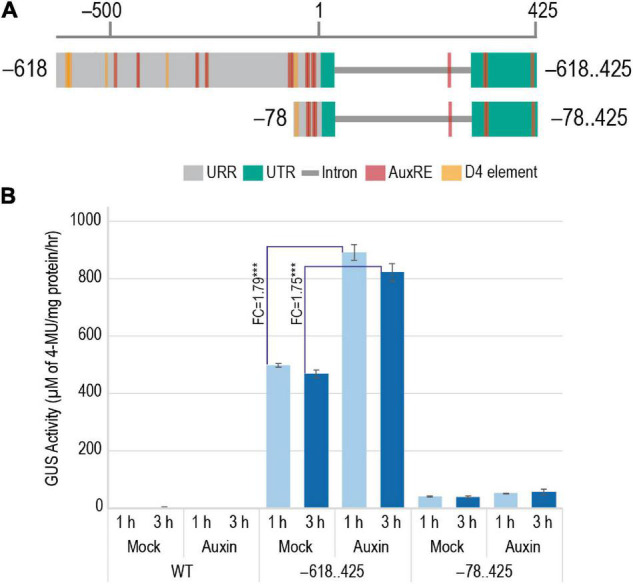
Effect of addition of exogenous auxin on the GUS activity in 7-day-old WT and transgenic plants harboring –618..425:pMDC164 and –78..425:pMDC164 constructs. **(A)** Diagrammatic representation of the –618..425 and –78..425 regions showcasing the auxin-responsive elements, AuxRE and D4 elements. **(B)** A bar graph showing GUS activity as μM of 4-MU/mg protein/h in response to exogenous IAA- and mock-treated plants. Fold changes in GUS activity between mock and auxin-induced samples for –618..425:pMDC164 construct have been indicated. Data represent information obtained from three biological replicates. Three asterisks represent Student’s *t*-test *p*-value < 0.001, and standard error is represented as error bars.

An earlier study by Yin and Xue in 2013 indicated the involvement of auxin in the transcriptional regulation of M29. In their study, WT ZH11 ovaries were cultured on media with and without exogenous auxin to show that the expression of *M29* increased in the ovaries cultured on auxin-supplemented media. To identify the *cis*-elements involved in the auxin-mediated induction of M29 expression, we tested 7-day-old T_3_ generation transgenic seedlings harboring –618..425 and –78..425 constructs (Figure 10A) along with WT untransformed seedlings for the induction of GUS activity after exogenous application of IAA. The –355..425 construct was not included in this study because the GUS expression profile in these lines was highly similar to those with the –618..425 construct. The –78..425 construct was used as a control in this study as it is unable to drive reporter expression like the –618 and –355 constructs. For this analysis, 7-day-old T_3_ transgenic and WT untransformed control seedlings were grown hydroponically in controlled conditions and treated with 50 μM IAA (Jain and Khurana, 2009) supplemented in Rice Growth Medium (RGM) (Yoshida et al., 1976) and incubated for 1 and 3 h. Mock-treated samples were used as controls. GUS activity post treatment was measured in terms of accumulation of 4-methylumbelliferone (4-MU), μM of 4-MU/mg protein/h. The GUS activity observed in mock and auxin-induced samples is depicted in Figure 10B. Three biological replicates were taken for each sample in the study. As expected, the WT did not show any GUS activity. The –78..425 construct showed minor induction by IAA. However, –618..425 samples showed a significant induction of 1.79 fold (*p*-value < 0.001) after 1 h and 1.75 fold (*p*-value < 0.001) 3 h after IAA treatment. The levels decline after 3 h, probably due to supraoptimal levels of auxin in the system. Our data indicate that the auxin-responsive *cis*-elements in the M29 URR from –78 to –618 region are functional and responsive to auxin and may be involved in auxin-mediated transcriptional regulation of M29.

### Characterization of a Distal Negative Element

**FIGURE 11:**
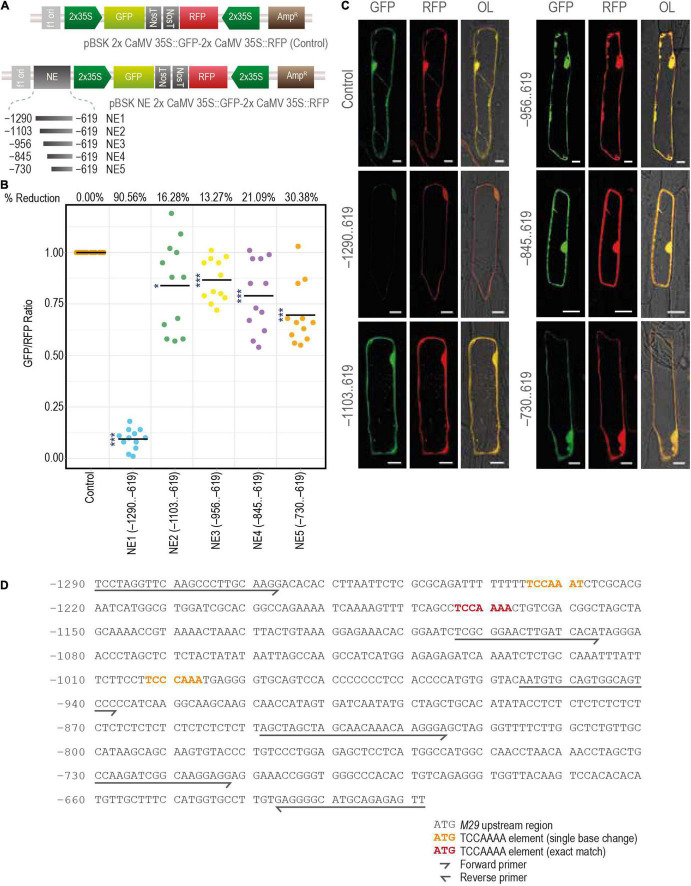
Characterization of the distal negative element. **(A)** The constructs used for the bombardment in the onion peel cells. **(B)** The scatter plot constructed using the online tool PlotsOfData (https://huygens.science.uva.nl/PlotsOfData/) shows the measured GFP/RFP ratio of the control vector containing both the GFP and RFP under same but individual 2x CaMV 35S promoter and the vectors containing the sequential deletions of the negative element region, from –1290..–619. The readings of the control vector samples were normalized to 1, and for the other vectors, the values were scaled accordingly. The percentage reduction is mentioned at the top of the graph for each corresponding construct. Here, *N* = 12, the asterisks represent Student’s *t*-test *p*-value, *** represents < 0.001, and * represents between 0.05 and 0.01. **(C)** Fluorescence images of onion peel cells bombarded with the mentioned constructs. Samples were visualized and photographed using the Leica SP5 confocal laser scanning microscope. Here, GFP, green fluorescent protein; RFP, red fluorescent protein; NE, negative element; and OL, overlay; the scale bar represents 25 μm. **(D)** The sequence of *M29* URR from –1290..–619 used for the identification of negative elements showing the TCCAAAA motifs.

As described above, GUS activity was not observed in any of the tissues at any developmental stage when the –1290..425 region of the URR was used to drive the expression of the downstream GUS reporter gene in transgenic rice plants. However, deletion of the –1290..–619 region restored the promoter activity, resulting in GUS expression in both vegetative and reproductive tissues. These data suggest the presence of a distal negative regulatory domain/element in the –1290..–619 region. To understand the nature and strength of this putative negative regulatory domain, we analyzed the effect of the –1290..–619 (NE1) region and its four serial deletions, namely, –1103..–619 (NE2), –956..–619 (NE3), –845..–619 (NE4), and –730..–619 (NE5), on the activity of a heterologous (2x Cauliflower Mosaic Virus 35S; 2x CaMV 35S) promoter driving a GFP (mGFP) CDS. The expression of RFP driven by another 2x CaMV 35S promoter in the same vector backbone was used as a control (Figure 11A and Supplementary Figures 3A,B). These reporter constructs were transiently expressed in onion peel cells by particle bombardment, and the ratios of fluorescence intensities (as measured under a confocal laser scanning microscope) of both the reporters (GFP/RFP) were taken as the measure of the strength of the negative element. The results of these experiments showed that the NE1 region was able to downregulate the activity of the 2x CaMV 35S promoter by 90.56% (Figure 11B). However, in the case of the other four serial deletion constructs (NE2, NE3, NE4, and NE5) the downregulation in GFP expression was limited to 16.28, 13.27, 21.09, and 30.38%, respectively (Figures 11B,C). These results are suggestive of the presence of a strong NE in the 186 base pair region between –1,290 and –1,104, while minor NE activities may exist in the region spanning between –1,104 and –619 nt in the M29 URR.

We also searched previously characterized negative regulatory *cis*-elements, including the GTACT motif reported in the moss system, *Barbula unguiculata* (Nagae et al., 2008), a TCCAAAA motif identified in watermelon (Yin et al., 2008), and a TGTGAGAGA motif reported in *Arabidopsis* (Simon et al., 2012) within the –1,290,–619 MADS29 URR. We identified three copies of the TCCAAAA motif in this region, of which two copies are located between –1,290 and –1,105 (one exact match and the other with only a single base change from A to T at the 7th position), and the third between –1,104 and –957 (Figure 11D). The third site also shows a single base difference at the 4th position (A to C). Although this element has been characterized in watermelon, it would be interesting to see if the same motif can downregulate the activity of the downstream promoter.

## Discussion

The transition from gametophytic to sporophytic phase leading to seed development after fertilization constitutes a landmark regulatory switch in the life cycle of haplodiplontic plants. This process involves a paradigm shift in gene expression patterns of hundreds of genes in a developmentally, temporally, and spatially regulated manner during the early stages of seed development (Sharma et al., 2012; Chen et al., 2014; Ma et al., 2019; Yi et al., 2019). Understanding the workings of regulatory components such as transcription factors and signal transduction components and their interactions with environmental and hormonal cues is the key to developing strategies for augmenting yield and nutritional value in cereal grains. M29 is a transcriptional factor that regulates starch biosynthesis during endosperm development in rice. It has also been found to be involved in the degradation of the nucellus and nucellar projection by regulating PCD (Yin and Xue, 2012). M29 also plays a role in embryo and endosperm development, as suppression of M29 expression by RNAi leads to wide-ranging deformities in embryos in these plants, preventing the seeds from germination (Nayar et al., 2013). Furthermore, M29 has also been implicated in shifting the auxin/cytokinin balance in favor of cytokinin. Probably for being involved in such vital aspects of postfertilization seed development, nature has devised complex and multiple-level controls for M29 expression.

M29 has been shown to express itself very specifically in the gynoecium of mature flowers and in different tissues of developing seeds at other time points. In seeds, the expression of M29 first appears in the dorsal vein in the 0–4 DAP period, and then the protein is shown to accumulate in the nucellar notch, followed by the outer cell layers of the endosperm. In the embryo, the M29 protein begins to accumulate from 6 DAP (Yin and Xue, 2012; Nayar et al., 2013). In this study, we analyzed the 1,715 bp URR spanning between –1,290 and + 425 nt of the M29 locus to delineate *cis*-elements that contribute to developmental and spatial gene expression patterns during the early stages of seed development in rice. We designed four deletion constructs of the M29 URR, i.e., –1290..425, –618..425, –355..425, and –78..425, in combination with the GUS reporter gene in the pMDC164 backbone.

Interestingly, we did not detect any GUS expression when the largest URR construct (–1290..425) was used. However, removing the distal 672 bp (i.e., from –1,290 to –619 bp) leads to robust GUS expression in both vegetative and reproductive tissues. This indicated the presence of a strong negative region in the distal promoter region. In seeds, the expression was found to mimic the RNA *in situ* and M29-immunolocalization profiles as the expression was restricted to the dorsal vein, embryo, and outer layers of the endosperm. However, non-specific GUS expression in leaves, roots, and floral glumes was also observed. This pattern of expression in vegetative and reproductive tissues continued even in the smaller –355..425 construct. However, in the case of the –355..425 construct, the GUS expression was restricted to the dorsal and ventral veins and outer endosperm cell layers, as the embryo was devoid of any GUS expression in the early stages (until 14 DAP). Finally, the –78..425 URR activity was restricted to the inner cell layers of the endosperm, while minimal GUS activity was also observed in vegetative tissues. This could be a result of the endosperm-specific motifs present in the 5′-UTR of M29. Studies have shown non-specific GUS expression in vegetative tissues of promoter:GUS transgenic plants arising due to interference from a nearby CaMV 35S promoter (Yoo et al., 2005). The GUS expression observed in the vegetative tissues in our study could also result from such interference by a strong CaMV 35S promoter present within the pMDC164 T-DNA region, i.e., being used to drive the antibiotic resistance gene (Figure 2A).

### Indole-3-Acetic Acid Induces M29 Expression

**FIGURE 12:**
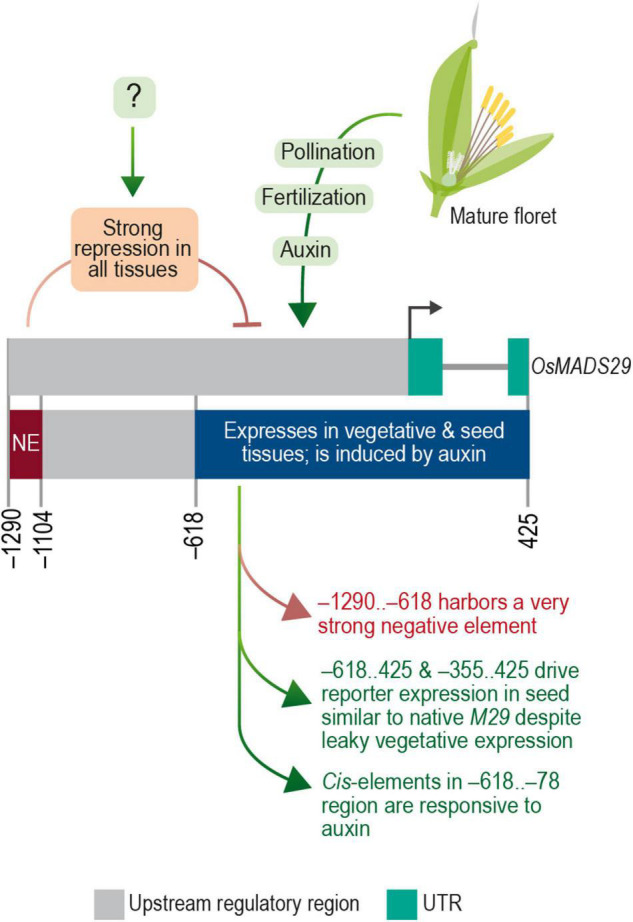
The transcriptional regulatory module of M29. Diagrammatic representation of transcription regulatory domains of OsMADS29 identified in this study.

Various research groups have highlighted the role of auxin in embryo and endosperm development in both monocot and dicot model systems (Friml et al., 2003; Uchiumi and Okamoto, 2010; Abu-Zaitoon et al., 2012; Chen et al., 2014; French et al., 2014). The auxin biosynthesis-related genes are upregulated in developing rice embryos as early as 1 DAP (Uchiumi and Okamoto, 2010). In 2012, Yin and Xue provided evidence for induction of M29 expression by real-time PCR (RT-PCR) on WT-ZH11 ovaries supplied with exogenous IAA and 2,4-dichlorophenoxyacetic acid (2,4-D). In this study, we found several auxin-responsive elements in the M29 URR. To validate their functional response to auxin stimulus, the effect of exogenous IAA was analyzed on 7-day-old transgenic seedlings harboring –618..425 and –78..425 constructs. Our analysis revealed a significant increase in GUS activity in the –618..425 construct of 1.79 and 1.75 fold after 1 and 3 h after exposure to 50 μM IAA as opposed to the slight induction from the minimal –78..425 construct. Our data are consistent with the auxin-based induction of M29 expression reported by Yin and Xue in 2012 and demonstrate that the *cis*-elements, which we predicted between the –618..425 nt region, are functionally responsive to IAA (Figure 12). This further implies that the AuxREs in the region upstream of –78 are more effective in making the promoter more responsive to auxins. However, a detailed analysis of the predicted *cis*-elements by mutating the elements or making further deletions in this region coupled with a yeast one-hybrid screen would reveal the precise motifs and their corresponding protein partners which are involved in the auxin responsiveness of M29 expression.

### Presence of Distal Negative Element(s) in M29 Upstream Regulatory Region

Negative or repressive elements may serve the purpose of preventing the illegitimate expression of crucial genes in undesirable cell/tissue types. The repression may be enforced or negated by way of a *trans*-factor acting downstream of a secondary messenger. For example, multiple GTACT motifs between –318 and –199 confer transcriptional repression of the iron superoxide dismutase (FeSOD) gene in response to copper in a moss, *Barbula unguiculata* (Nagae et al., 2008). Similarly, the proximal (from –472 to –424) and distal negative elements (from –986 to –959) harboring the TCCAAAA motif regulate seed-specific expression of the ADP-glucose pyrophosphorylase gene in watermelon by inhibiting its expression in leaves (Yin et al., 2008).

Our M29-URR:GUS fusion data revealed the presence of a putative repressor region between the –1,290 and –619 nt positions. Experiments involving dual promoter constructs driving GFP and RFP in the same vector backbone and placing four deletions (–1,103..–619, –956..–619, –845..–619, and –730..–619) of the putative negative regulatory region upstream of the promoter driving GFP helped in the further delineation of this region. These experiments helped in narrowing down the most effective negative regulatory region to a 186 bp region between –1,290 and –1,104 bases upstream of the M29 TSS. Similar approaches have been used in the past to ascertain the specific response of promoter regions and *cis*-elements appended to either a minimal or a complete 35S CaMV promoter with either luciferase or GUS reporter genes (Liu et al., 1990, 2020; Quayle et al., 1991; Treuter et al., 1993; Morita et al., 1998; Ishige et al., 1999).

Taken together, these data are indicative of a complex and modular architecture of the M29 URR, which includes (a) a distal negative region within the –1,290 to –619 nt region that suppresses the expression of M29 in both vegetative and reproductive tissues, (b) an auxin-responsive region within the –618 to –79 nt, and (c) the –78 to 425 nt region exhibiting minimal promoter activity along with expression specificity for the inner endosperm region. Our data also suggest that a *cis*-element(s) should exist to negate the repressive effect of the –1,290..–619 region in a seed-specific manner. Earlier, the GUS expression was observed in seeds and MFs in a similar experiment in which a –2.8 kb (∼2.35 upstream from TSS) URR region was analyzed for its promoter activity (Yin and Xue, 2012). The observed GUS activity in seeds with a longer URR is suggestive of an element within the –2,350 and –1,290 regions that can negate the repressive effect of the –1,290..–619 region. However, the observed GUS activity in floral tissues in the case of the −2.8 kb URR leaves scope for further investigation to delineate the promoter elements that contribute to the specificity of M29 URR in seed tissues. Taken together, both studies showcase the complex regulation of *MADS29* expression in rice. The summary of our findings is depicted in Figure 12.

## Conclusion

The data presented here point to a complex interplay of negative, positive, and auxin-responsive *cis*-elements in the URR of a transcription factor-encoding gene that regulates vital aspects of seed development in rice. The insights obtained from this study might therefore hint toward the possible existence of a similar arrangement of *cis*-regulatory modules in many other genes involved in early seed development in monocots.

## Data Availability Statement

The original contributions presented in the study are included in the article/Supplementary Material, further inquiries can be directed to the corresponding author/s.

## Author Contributions

RK, SB, KR, MK, and SK conceptualized the study. RK, SB, and GS carried out the experiments. RK, SB, and SK wrote the manuscript. GS, KR, VV, NB, and GG contributed toward finalizing data and manuscript writing. All authors contributed to the article and approved the submitted version.

## Conflict of Interest

The authors declare that the research was conducted in the absence of any commercial or financial relationships that could be construed as a potential conflict of interest.

## Publisher’s Note

All claims expressed in this article are solely those of the authors and do not necessarily represent those of their affiliated organizations, or those of the publisher, the editors and the reviewers. Any product that may be evaluated in this article, or claim that may be made by its manufacturer, is not guaranteed or endorsed by the publisher.

## Supplementary Material

Supplementary Figure 1 Cloning of M29 URR deletions. **(I)** Strategy for cloning M29 URR deletions in pMDC164 vector. **(II)** Here **(A–C)** M29 –1290..425, **(D–F)** M29 –618..425, **(G–I)** M29 –355..425, **(J–L)** M29 –78..425 PCR amplified and cloned into pENTR/D-TOPO entry vector and after mobilized into plant transformation Gateway destination vector pMDC164 resulting in the fusion of M29 URR inserts with a downstream GUS reporter, respectively. The identity of inserts was validated by restriction digestion using the appropriate pair of enzymes as mentioned on top of each lane.

Supplementary Figure 2 Transformation of rice with pMDC164 constructs. **(A)** Diagrammatic representation of promoter-GUS fusion constructs in pMDC164 vector used for transformation of rice, highlighting the T-DNA region harboring the URR deletion inserts fused to a downstream GUS reporter and the CaMV 35S promoter-driven hygromycin antibiotic-selection gene. **(B–F)** Representative images of the stages of rice transformation; **(B)** embryonic half of mature rice seed inoculated in callus induction N6D medium, **(C)** 6-day old callus, **(D)** 21-day old callus showing white proliferating transgenic calli, **(E)** regenerating transgenic shoots, **(F)** plantlets with roots after 2-week induction of rooting on HF medium, and **(G)** mature transgenic plants.

Supplementary Figure 3A Cloning strategy for characterization of negative element. Strategy for cloning of **(A)** the control construct and **(B)** the NE and its deletions harboring constructs.

Supplementary Figure 3B Cloning of negative element constructs. **(A)** 2x CaMV 35S was PCR amplified and cloned in the pENTR/TOPO vector. It was then transferred to pMDC110 and pGWB653 *via* gateway cloning. **(B)** Cloning of 2x CaMV 35S:GFP:T cassette in the pBSK backbone *via* unidirectional restriction digestion-based method. **(C)** PCR amplification of 2x CaMV 35S:RFP:T with restriction enzyme sites in the primers and mobilizing the amplicon in pBSK:2x CaMV 35S:GFP:T vector by bidirectional restriction digestion based cloning. **(D)** PCR amplification of the NE1, NE2, NE3, NE4, and NE5 regions with *Not*I restriction enzyme sites in the primer and mobilizing it in pBSK:2x CaMV 35S:GFP:T_2x CaMV 35S:RFP:T vector *via* bidirectional restriction digestion based cloning.

## References

[B1] Abiko M. Maeda H. Tamura K. Hara-Nishimura I. Okamoto T. (2013). Gene expression profiles in rice gametes and zygotes: identification of gamete-enriched genes and up- or down-regulated genes in zygotes after fertilization. *J. Exp. Bot.* 64 1927–1940. 10.1093/jxb/ert054 23570690 PMC3638821

[B2] Abu-Zaitoon Y. M. Bennett K. Normanly J. Nonhebel H. M. (2012). A large increase in IAA during development of rice grains correlates with the expression of tryptophan aminotransferase OsTAR1 and a grain-specific YUCCA. *Physiol. Plant* 146 487–499. 10.1111/j.1399-3054.2012.01649.x 22582989

[B3] Anderson S. N. Johnson C. S. Chesnut J. Jones D. S. Khanday I. Woodhouse M. (2017). The zygotic transition is initiated in unicellular plant zygotes with asymmetric activation of parental genomes. *Dev. Cell* 43 349–358.e4. 10.1016/j.devcel.2017.10.005 29112853

[B4] Arul L. Benita G. Sudhakar D. Balasubramanian B. T. Balasubramanian P. (2008). β-glucuronidase of family-2 glycosyl hydrolase: a missing member in plants. *Bioinformation* 3 194–197. 10.6026/97320630003194 19255633 PMC2646188

[B5] Berendzen K. W. Weiste C. Wanke D. Kilian J. Harter K. Dröge-Laser W. (2012). Bioinformatic cis-element analyses performed in *Arabidopsis* and rice disclose bZIP- and MYB-related binding sites as potential AuxRE-coupling elements in auxin-mediated transcription. *BMC Plant Biol.* 12:125. 10.1186/1471-2229-12-125 22852874 PMC3438128

[B6] Borah P. Sharma E. Kaur A. Chandel G. Mohapatra T. Kapoor S. (2017). Analysis of drought-responsive signalling network in two contrasting rice cultivars using transcriptome-based approach. *Sci. Rep.* 7:42131. 10.1038/srep42131 28181537 PMC5299611

[B7] Borg M. Papareddy R. K. Dombey R. Axelsson E. Nodine M. D. Twell D. (2021). Epigenetic reprogramming rewires transcription during the alternation of generations in Arabidopsis. *eLife* 10:e61894. 10.7554/eLife.61894 33491647 PMC7920552

[B8] Broeckling B. E. Watson R. A. Steinwand B. Bush D. R. (2016). Intronic sequence regulates sugar-dependent expression of arabidopsis thaliana production of anthocyanin pigment-1/MYB75. *PLoS One* 11:e0156673. 10.1371/journal.pone.0156673 27248141 PMC4889055

[B9] Chen J. Zeng B. Zhang M. Xie S. Wang G. Hauck A. (2014). Dynamic transcriptome landscape of maize embryo and endosperm development. *Plant Physiol.* 166 252–264. 10.1104/pp.114.240689 25037214 PMC4149711

[B10] Cherenkov P. Novikova D. Omelyanchuk N. Levitsky V. Grosse I. Weijers D. (2018). Diversity of cis-regulatory elements associated with auxin response in *Arabidopsis thaliana*. *J. Exp. Bot.* 69 329–339. 10.1093/jxb/erx254 28992117 PMC5853796

[B11] Coruzzi G. Broglie R. Edwards C. Chua N. H. (1984). Tissue-specific and light-regulated expression of a pea nuclear gene encoding the small subunit of ribulose-1,5-bisphosphate carboxylase. *EMBO J.* 3 1671–1679. 10.1002/j.1460-2075.1984.tb02031.x 6479146 PMC557581

[B12] Curtis M. D. Grossniklaus U. (2003). A gateway cloning vector set for high-throughput functional analysis of genes in planta. *Plant Physiol.* 133 462–469. 10.1104/pp.103.027979 14555774 PMC523872

[B13] Deushi R. Toda E. Koshimizu S. Yano K. Okamoto T. (2021). Effect of paternal genome excess on the developmental and gene expression profiles of polyspermic zygotes in rice. *Plants* 10:255. 10.3390/plants10020255 33525652 PMC7911625

[B14] Deveshwar P. Bovill W. D. Sharma R. Able J. A. Kapoor S. (2011). Analysis of anther transcriptomes to identify genes contributing to meiosis and male gametophyte development in rice. *BMC Plant Biol.* 11:78. 10.1186/1471-2229-11-78 21554676 PMC3112077

[B15] Foster R. Takeshi I. Chua N. H. (1994). Plant bZIP proteins gather at ACGT elements. *FASEB J.* 8 192–200. 10.1096/fasebj.8.2.8119490 8119490

[B16] French S. R. Abu-Zaitoon Y. Uddin M. M. Bennett K. Nonhebel H. M. (2014). Auxin and cell wall invertase related signaling during rice grain development. *Plants* 3 95–112. 10.3390/plants3010095 27135493 PMC4844310

[B17] Friml J. Vieten A. Sauer M. Weijers D. Schwarz H. Hamann T. (2003). Efflux-dependent auxin gradients establish the apical–basal axis of Arabidopsis. *Nature* 426 147–153. 10.1038/nature02085 14614497

[B18] Higo K. (1998). PLACE: a database of plant cis-acting regulatory DNA elements. *Nucleic Acids Res.* 26 358–359. 10.1093/nar/26.1.358 9399873 PMC147199

[B19] Ichihashi Y. Hakoyama T. Iwase A. Shirasu K. Sugimoto K. Hayashi M. (2020). Common mechanisms of developmental reprogramming in plants—lessons from regeneration, symbiosis, and parasitism. *Front. Plant Sci.* 11:1084. 10.3389/fpls.2020.01084 32765565 PMC7378864

[B20] Ishige F. Takaichi M. Foster R. Chua N.-H. Oeda K. (1999). A G-box motif (GCCACGTGCC) tetramer confers high-level constitutive expression in dicot and monocot plants. *Plant J.* 18 443–448. 10.1046/j.1365-313x.1999.00456.x

[B21] Itoh J. Sato Y. Sato Y. Hibara K. Shimizu-Sato S. Kobayashi H. (2016). Genome-wide analysis of spatio-temporal gene expression patterns during early embryogenesis in rice. *Development* 143 1217–1227. 26903508 10.1242/dev.123661

[B22] Jain M. Khurana J. P. (2009). Transcript profiling reveals diverse roles of auxin-responsive genes during reproductive development and abiotic stress in rice. *FEBS J.* 276 3148–3162. 10.1111/j.1742-4658.2009.07033.x 19490115

[B23] Jefferson R. A. (1987). Assaying chimeric genes in plants: the GUS gene fusion system. *Plant Mol. Biol. Rep.* 5 387–405. 10.1007/bf02667740

[B24] Kuhlemeier C. Fluhr R. Green P. J. Chua N. H. (1987). Sequences in the pea rbcS-3A gene have homology to constitutive mammalian enhancers but function as negative regulatory elements. *Genes Dev.* 1 247–255. 10.1101/gad.1.3.247 2890552

[B25] Lavy M. Estelle M. (2016). Mechanisms of auxin signaling. *Development* 143 3226–3229. 10.1242/dev.131870 27624827 PMC5047657

[B26] Lee L.-Y. Fang M.-J. Kuang L.-Y. Gelvin S. B. (2008). Vectors for multi-color bimolecular fluorescence complementation to investigate protein-protein interactions in living plant cells. *Plant Methods* 4:24. 10.1186/1746-4811-4-24 18922163 PMC2572157

[B27] Liu R. Zou X. Wang Y. Long Q. Pei Y. (2020). A 100 bp GAGA motif-containing sequence in AGAMOUS second intron is able to suppress the activity of CaMV35S enhancer in vegetative tissues. *PLoS One* 15:e0230203. 10.1371/journal.pone.0230203 32134990 PMC7058354

[B28] Liu X. J. Prat S. Willmitzer L. Frommer W. B. (1990). Cis regulatory elements directing tuber-specific and sucrose-inducible expression of a chimeric class I patatin promoter/GUS-gene fusion. *Mol. Gen. Genet.* 223 401–406. 10.1007/BF00264446 2270080

[B29] Liu Z. Yuan G. Liu S. Jia J. Cheng L. Qi D. (2017). Identified of a novel cis-element regulating the alternative splicing of LcDREB2. *Sci. Rep.* 7:46106. 10.1038/srep46106 28383047 PMC5382683

[B30] Ma C. Li B. Wang L. Xu M. Lizhu E. Jin H. (2019). Characterization of phytohormone and transcriptome reprogramming profiles during maize early kernel development. *BMC Plant Biol.* 19:197. 10.1186/s12870-019-1808-9 31088353 PMC6515667

[B31] Mironova V. V. Omelyanchuk N. A. Wiebe D. S. Levitsky V. G. (2014). Computational analysis of auxin responsive elements in the *Arabidopsis thaliana* L. genome. *BMC Genomics* 15:S4. 10.1186/1471-2164-15-S12-S4 25563792 PMC4331925

[B32] Morita A. Umemura T. Kuroyanagi M. Futsuhara Y. Perata P. Yamaguchi J. (1998). Functional dissection of a sugar-repressed K-amylase gene (RAmy1A) promoter in rice embryos. *FEBS Lett.* 423 81–85. 10.1016/s0014-5793(98)00067-2 9506846

[B33] Nagae M. Nakata M. Takahashi Y. (2008). Identification of negative cis-acting elements in response to copper in the chloroplastic iron superoxide dismutase gene of the moss *Barbula unguiculata*. *Plant Physiol.* 146 1687–1696. 10.1104/pp.107.114868 18258690 PMC2287343

[B34] Nayar S. Kapoor M. Kapoor S. (2014). Post-translational regulation of rice MADS29 function: homodimerization or binary interactions with other seed-expressed MADS proteins modulate its translocation into the nucleus. *J. Exp. Bot.* 65 5339–5350. 10.1093/jxb/eru296 25096923 PMC4157715

[B35] Nayar S. Sharma R. Tyagi A. K. Kapoor S. (2013). Functional delineation of rice MADS29 reveals its role in embryo and endosperm development by affecting hormone homeostasis. *J. Exp. Bot.* 64 4239–4253. 23929654 10.1093/jxb/ert231 PMC3808311

[B36] Nemhauser J. L. Hong F. Chory J. (2006). Different plant hormones regulate similar processes through largely nonoverlapping transcriptional responses. *Cell* 126 467–475. 10.1016/j.cell.2006.05.050 16901781

[B37] Onodera Y. Suzuki A. Wu C.-Y. Washida H. Takaiwa F. (2001). A rice functional transcriptional activator. RISBZ1, responsible for endosperm-specific expression of storage protein genes through GCN4 motif. *J. Biol. Chem.* 276 14139–14152. 10.1074/jbc.M007405200 11133985

[B38] Postma M. Goedhart J. (2019). PlotsOfData—A web app for visualizing data together with their summaries. *PLoS Biol.* 17:e3000202. 10.1371/journal.pbio.3000202 30917112 PMC6453475

[B39] Powers S. K. Strader L. C. (2020). Regulation of auxin transcriptional responses. *Dev. Dyn.* 249 483–495. 10.1002/dvdy.139 31774605 PMC7187202

[B40] Qu L. Q. Xing Y. P. Liu W. X. Xu X. P. Song Y. R. (2008). Expression pattern and activity of six glutelin gene promoters in transgenic rice. *J. Exp. Bot.* 59 2417–2424. 10.1093/jxb/ern110 18467323 PMC2423653

[B41] Quayle T. J. A. Hetz W. Feix G. (1991). Characterization of a maize endosperm culture expressing zein genes and its use in transient transformation assays. *Plant Cell Rep.* 9 544–548. 10.1007/BF00232328 24220708

[B42] Rice Annotation Project (2007). The Rice Annotation Project Database (RAP-DB): 2008 update. *Nucleic Acids Res.* 36 D1028–D1033. 10.1093/nar/gkm978 18089549 PMC2238920

[B43] Sharma R. Agarwal P. Ray S. Deveshwar P. Sharma P. Sharma N. (2012). Expression dynamics of metabolic and regulatory components across stages of panicle and seed development in indica rice. *Funct. Integr. Genomics* 12 229–248. 10.1007/s10142-012-0274-3 22466020

[B44] She W. Grimanelli D. Rutowicz K. Whitehead M. W. J. Puzio M. Kotliński M. (2013). Chromatin reprogramming during the somatic-to-reproductive cell fate transition in plants. *Development* 140 4008–4019. 10.1242/dev.095034 24004947

[B45] Shlyueva D. Stampfel G. Stark A. (2014). Transcriptional enhancers: from properties to genome-wide predictions. *Nat. Rev. Genet.* 15 272–286. 10.1038/nrg3682 24614317

[B46] Simon M. K. Williams L. A. Brady-Passerini K. Brown R. H. Gasser C. S. (2012). Positive- and negative-acting regulatory elements contribute to the tissue-specific expression of INNER NO OUTER, a YABBY-type transcription factor gene in Arabidopsis. *BMC Plant Biol.* 12:214. 10.1186/1471-2229-12-214 23148487 PMC3583067

[B47] Simpson J. Schell J. Montagu M. V. Herrera-Estrella L. (1986). Light-inducible and tissue-specific pea lhcp gene expression involves an upstream element combining enhancer- and silencer-like properties. *Nature* 323 551–554. 10.1038/323551a0

[B48] Sudan C. Prakash S. Bhomkar P. Jain S. Bhalla-Sarin N. (2006). Ubiquitous presence of β-glucuronidase (GUS) in plants and its regulation in some model plants. *Planta* 224 853–864. 10.1007/s00425-006-0276-2 16652220

[B49] Tapia-Tussell R. Quijano-Ramayo A. Rojas-Herrera R. Larque-Saavedra A. Perez-Brito D. (2005). A fast, simple, and reliable high-yielding method for DNA extraction from different plant species. *Mol. Biotechnol.* 31 137–140. 10.1385/MB:31:2:137 16170214

[B50] Toki S. Hara N. Ono K. Onodera H. Tagiri A. Oka S. (2006). Early infection of scutellum tissue with Agrobacterium allows high-speed transformation of rice. *Plant J.* 47 969–976. 10.1111/j.1365-313X.2006.02836.x 16961734

[B51] Treuter E. Nover L. Ohme K. Scharf K.-D. (1993). Promoter specificity and deletion analysis of three heat stress transcription factors of tomato. *Mol. Gen. Genet.* 240 113–125. 10.1007/BF00276890 8341257

[B52] Uchiumi T. Okamoto T. (2010). Rice fruit development is associated with an increased IAA content in pollinated ovaries. *Planta* 232 579–592. 10.1007/s00425-010-1197-7 20512651

[B53] Ulmasov T. Liu Z.-B. Hagen G. Guilfoyle’ T. J. (1995). Composite structure of auxin response elements. *Plant Cell* 7 1161–1623. 10.1105/tpc.7.10.1611 7580254 PMC161020

[B54] Wang H.-L. V. Chekanova J. A. (2019). Novel mRNAs 3′ end-associated *cis* -regulatory elements with epigenomic signatures of mammalian enhancers in the *Arabidopsis* genome. *RNA* 25 1242–1258. 10.1261/rna.071209.119 31311821 PMC6800480

[B55] Weber B. Zicola J. Oka R. Stam M. (2016). Plant enhancers: a call for discovery. *Trends Plant Sci.* 21 974–987. 10.1016/j.tplants.2016.07.013 27593567

[B56] Wu L. Luo P. Di D. W. Wang L. Wang M. Lu C. K. (2015). Forward genetic screen for auxin-deficient mutants by cytokinin. *Sci. Rep.* 5:11923. 10.1038/srep11923 26143750 PMC4491711

[B57] Xie Y. Zhang Y. Han J. Luo J. Li G. Huang J. (2018). The intronic cis element SE1 recruits trans-acting repressor complexes to repress the expression of ELONGATED UPPERMOST INTERNODE1 in Rice. *Mol. Plant* 11 720–735. 10.1016/j.molp.2018.03.001 29524649

[B58] Yi F. Gu W. Chen J. Song N. Gao X. Zhang X. (2019). High temporal-resolution transcriptome landscape of early maize seed development. *Plant Cell* 31 974–992. 10.1105/tpc.18.00961 30914497 PMC6533015

[B59] Yin L.-L. Xue H.-W. (2012). The *MADS29* transcription factor regulates the degradation of the nucellus and the nucellar projection during rice seed development. *Plant Cell* 24 1049–1065. 10.1105/tpc.111.094854 22408076 PMC3336122

[B60] Yin T. Wu H. Zhang S. Liu J. Lu H. Zhang L. (2008). Two negative cis-regulatory regions involved in fruit-specific promoter activity from watermelon (*Citrullus vulgaris* S.). *J. Exp. Bot.* 60 169–185. 10.1093/jxb/ern273 19073962 PMC3071764

[B61] Yoo S. Y. Bomblies K. Yoo S. K. Yang J. W. Choi M. S. Lee J. S. (2005). The 35S promoter used in a selectable marker gene of a plant transformation vector affects the expression of the transgene. *Planta* 221 523–530. 10.1007/s00425-004-1466-4 15682278

[B62] Yoshida S. F. D. A. Cock J. H. Gomez K. A. (1976). *Laboratory Manual for Physiological Studies of Rice*, 3rd Edn. Los Baños: THE INTERNATIONAL RICE RESEARCH INSTITUTE.

