# Supplementary figures and images for: Characterization of Transcription Regulatory Domains of OsMADS29: Identification of Proximal Auxin-Responsive Domains and a Strong Distal Negative Element
Source: Front Plant Sci. 2022 Apr 25;13:850956. doi: 10.3389/fpls.2022.850956 (PMC9085466; doi:10.3389/fpls.2022.850956)

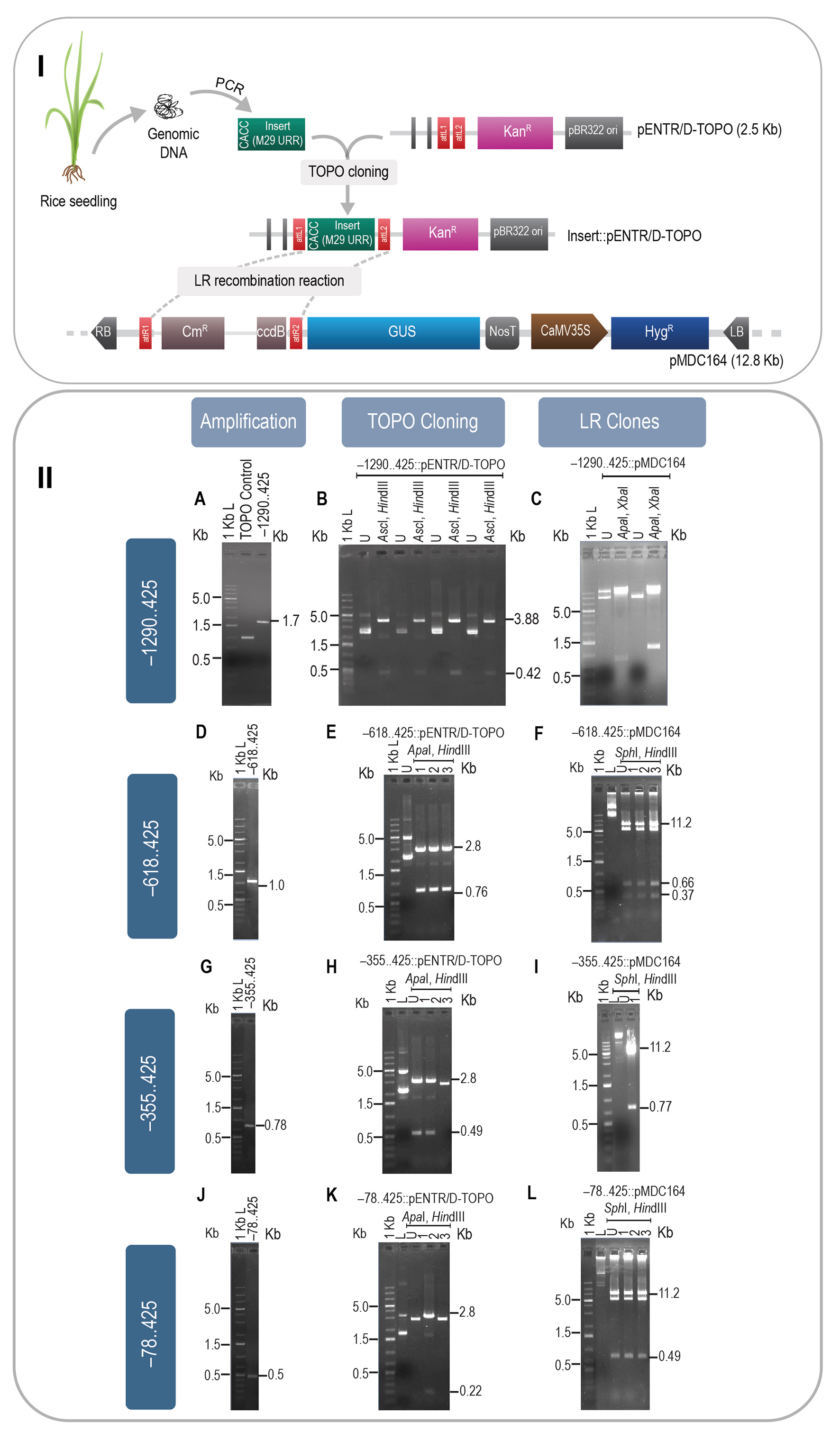

Supplement: Supplementary Figure 1 — Cloning of M29 URR deletions. (I) Strategy for cloning M29 URR deletions in pMDC164 vector. (II) Here (A–C) M29 –1290..425, (D–F) M29 –618..425, (G–I) M29 –355..425, (J–L) M29 –78..425 PCR amplified and cloned into pENTR/D-TOPO entry vector and after mobilized into plant transformation Gateway destination vector pMDC164 resulting in the fusion of M29 URR inserts with a downstream GUS reporter, respectively. The identity of inserts was validated by restriction digestion using the appropriate pair of enzymes as mentioned on top of each lane. [file Image_1.JPEG]

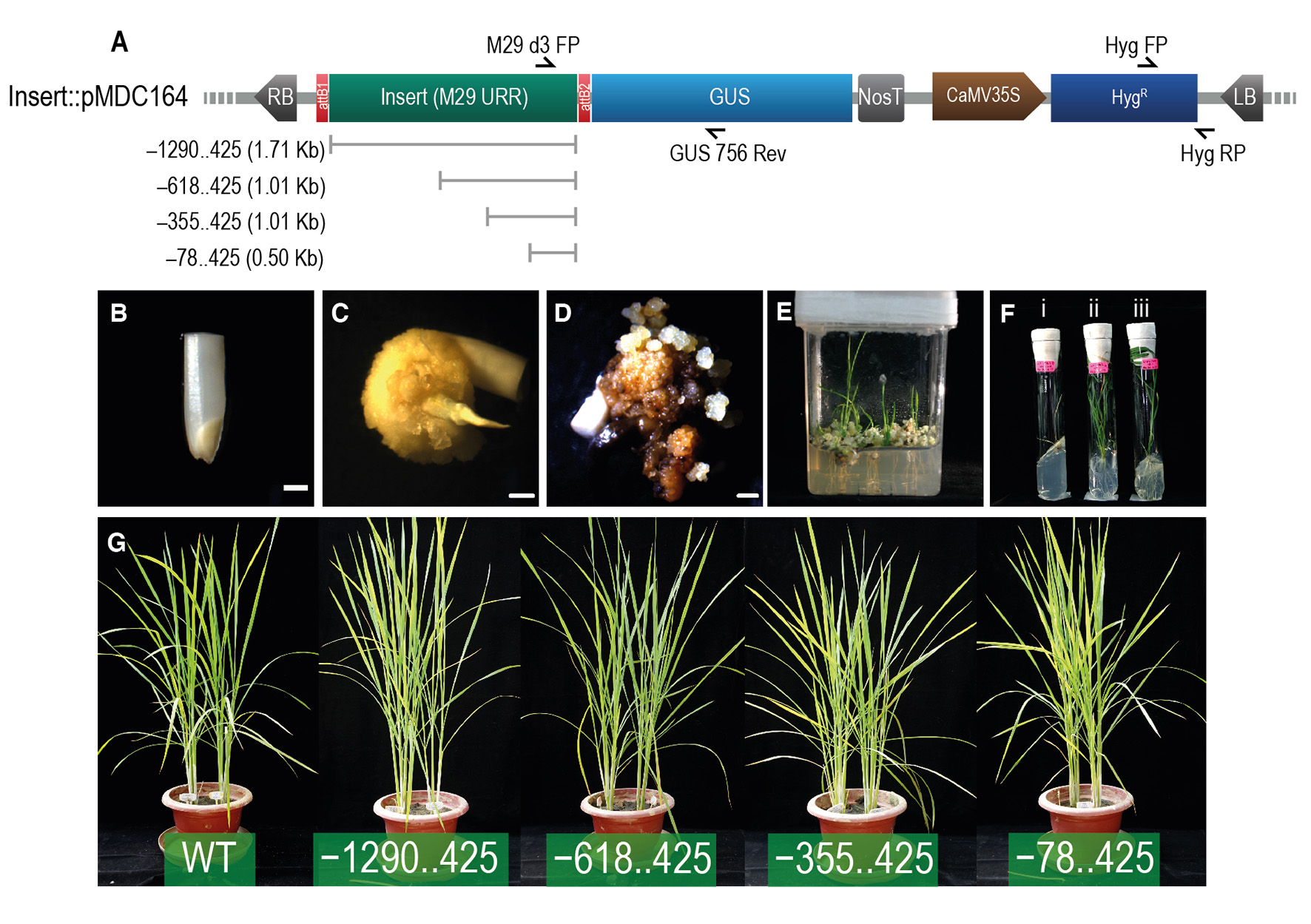

Supplement: Supplementary Figure 2 — Transformation of rice with pMDC164 constructs. (A) Diagrammatic representation of promoter-GUS fusion constructs in pMDC164 vector used for transformation of rice, highlighting the T-DNA region harboring the URR deletion inserts fused to a downstream GUS reporter and the CaMV 35S promoter-driven hygromycin antibiotic-selection gene. (B–F) Representative images of the stages of rice transformation; (B) embryonic half of mature rice seed inoculated in callus induction N6D medium, (C) 6-day old callus, (D) 21-day old callus showing white proliferating transgenic calli, (E) regenerating transgenic shoots, (F) plantlets with roots after 2-week induction of rooting on HF medium, and (G) mature transgenic plants. [file Image_2.JPEG]

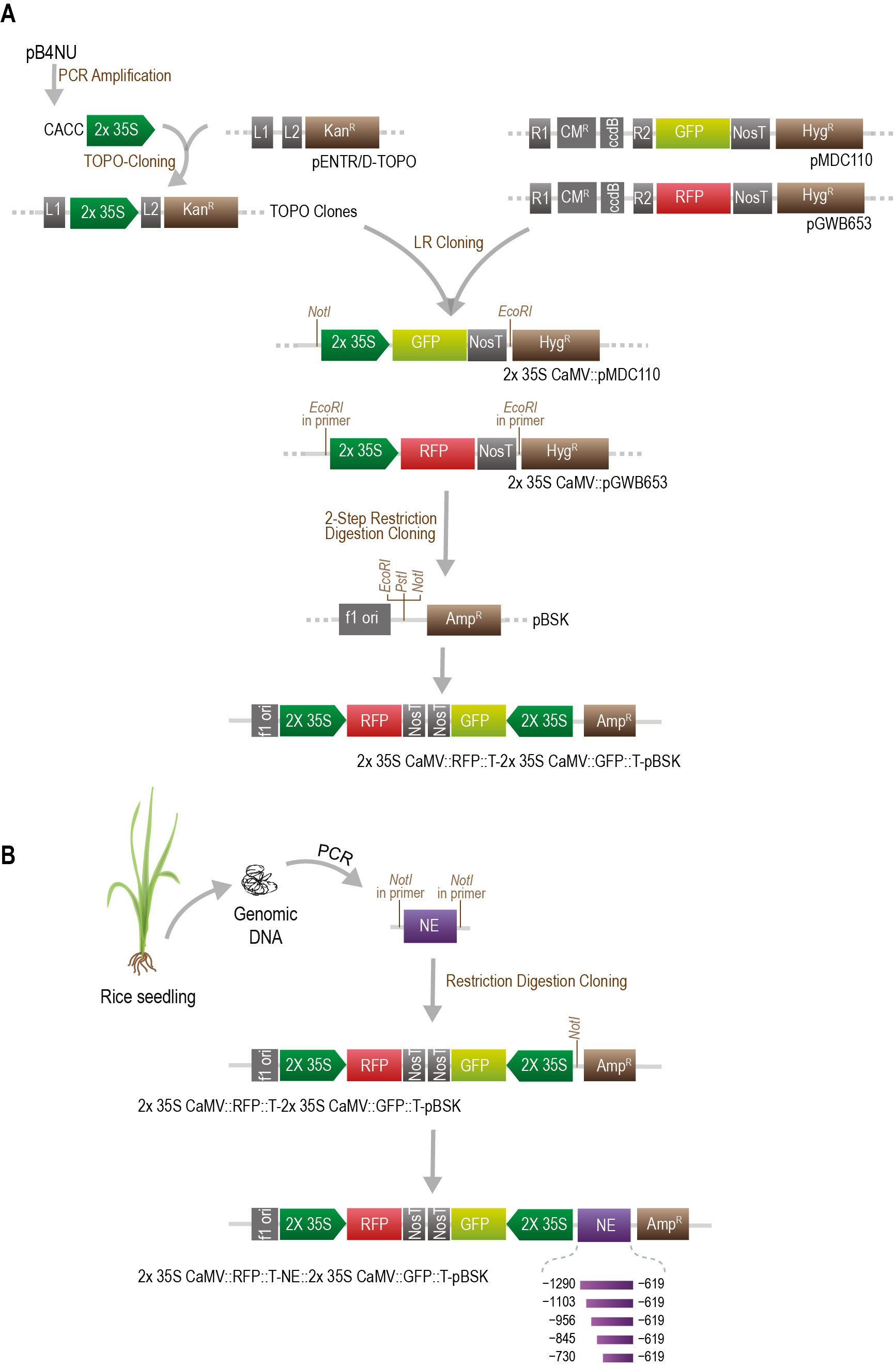

Supplement: Supplementary Figure 3A — Cloning strategy for characterization of negative element. Strategy for cloning of (A) the control construct and (B) the NE and its deletions harboring constructs. [file Image_3.JPEG]

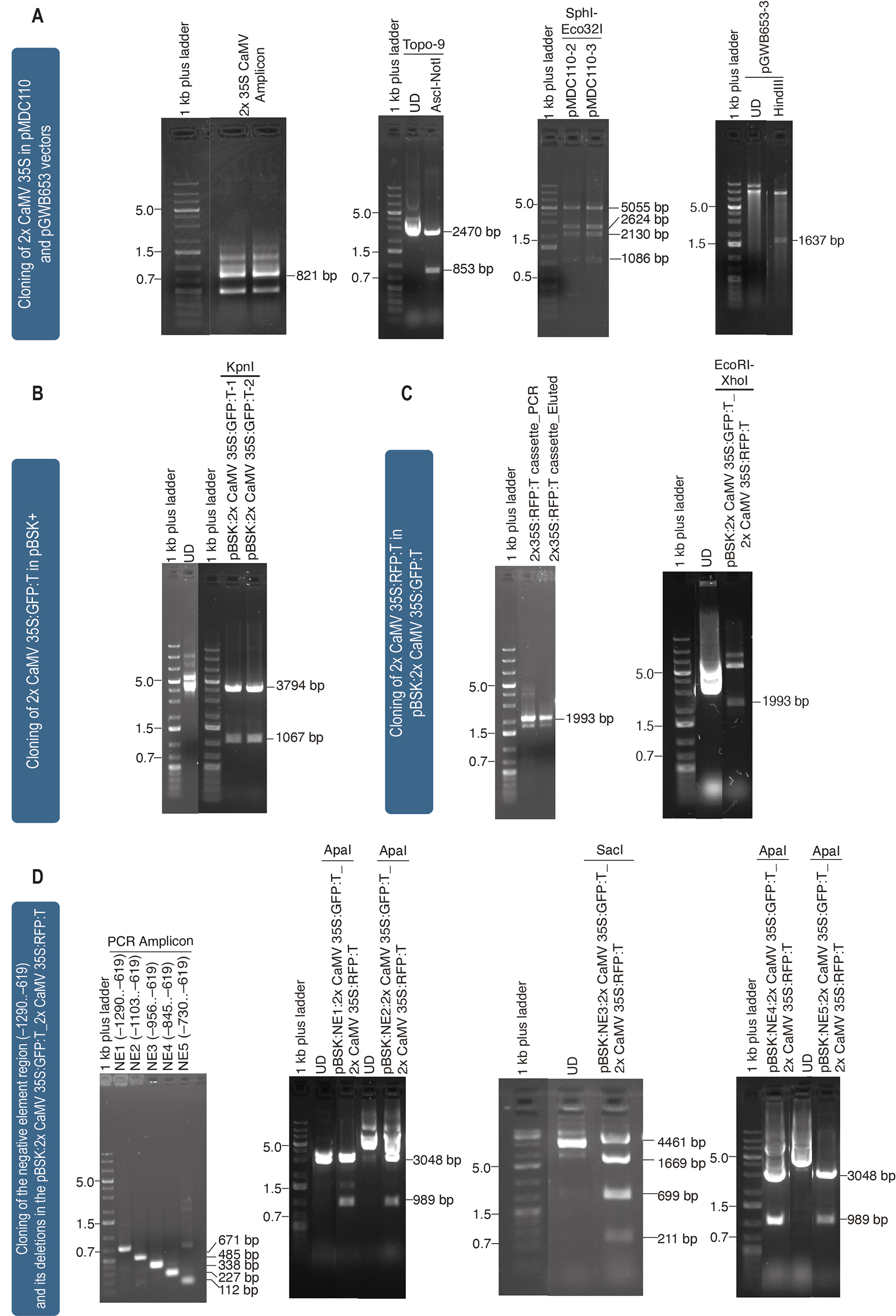

Supplement: Supplementary Figure 3B — Cloning of negative element constructs. (A) 2x CaMV 35S was PCR amplified and cloned in the pENTR/TOPO vector. It was then transferred to pMDC110 and pGWB653 via gateway cloning. (B) Cloning of 2x CaMV 35S:GFP:T cassette in the pBSK backbone via unidirectional restriction digestion-based method. (C) PCR amplification of 2x CaMV 35S:RFP:T with restriction enzyme sites in the primers and mobilizing the amplicon in pBSK:2x CaMV 35S:GFP:T vector by bidirectional restriction digestion based cloning. (D) PCR amplification of the NE1, NE2, NE3, NE4, and NE5 regions with NotI restriction enzyme sites in the primer and mobilizing it in pBSK:2x CaMV 35S:GFP:T_2x CaMV 35S:RFP:T vector via bidirectional restriction digestion based cloning. [file Image_4.JPEG]
